# The exploration and research on the establishment of an early risk assessment system for high-risk diabetic feet

**DOI:** 10.3389/fendo.2026.1819338

**Published:** 2026-06-15

**Authors:** Haixia Qi, Juan Li, Lijie Hou, Ruiping Huang, Lihua Ma

**Affiliations:** 1 School of Nursing, Lanzhou University, Lanzhou, China; 2 Department of Endocrinology, The First Hospital of Lanzhou University, Lanzhou, China

**Keywords:** Delphi method, high-risk diabetic foot, nursing prevention, risk assessment, system

## Abstract

**Purpose:**

To develop an early risk assessment system for high-risk diabetic feet.

**Methods:**

The system framework was established through literature review and two rounds of Delphi expert consultation.

**Results:**

The two rounds of consultation achieved effective response rates of 73.3% and 100.0%, with authority coefficients of 0.802 and 0.933, and Kendall’s coefficients of concordance (W) of 0.302 and 0.103 (all P < 0.05). Consensus was reached on an early risk assessment system comprising 6 dimensions (risk factor identification, neurological assessment, vascular evaluation, foot skin and nail examination, self-care ability assessment, and healthcare resource accessibility) and 42 items, enabling structured risk stratification for high-risk diabetic feet.

**Conclusion:**

The Delphi-validated system demonstrates good scientific rigor, rationality, practicality, and targetedness. This evidence-based tool can guide nursing training, standardize prevention and control practices, refine teaching programs, and evaluate institutional capabilities for high-risk diabetic foot management.

## 1 Introduction

The global prevalence of diabetes mellitus is steadily increasing, posing a substantial and growing public health burden. Epidemiological data from 2019 reported a global diabetes prevalence of 9.3%, corresponding to approximately 463 million affected individuals (1). Forecasts suggest that the prevalence will rise to 10.2% (570 million cases) by 2030 and further increase to 10.9% (over 700 million cases) by 2045. In China, the prevalence of diabetes among adults aged 18 years and above has reached 11.2%. As a major chronic disease, diabetes contributes to severe systemic complications, among which diabetic foot syndrome (DFS) is one of the most debilitating. DFS is characterized by foot ulcers, infections, and tissue destruction, usually accompanied by diabetic peripheral neuropathy (DPN) and peripheral artery disease (PAD) (2).

Epidemiological evidence shows that the global prevalence of diabetic foot (DF) is 6.30%, while the corresponding prevalence in China is 4.10% (3), with most cases concentrated in the elderly population aged over 60 years. More than 15% of people with diabetes will develop diabetic foot ulcers (DFUs) during their lifetime, of which over 50% progress to secondary infection. Foot infection is a leading cause of lower extremity amputation in diabetic populations (4). Without timely and standardized intervention, DFUs may develop into chronic non-healing wounds, further leading to foot deformities, limited joint mobility, muscle atrophy, recurrent bacterial infection, aggravated skin damage, and increased long-term disability rates (5). Notably, clinical evidence confirms that standardized early risk screening and targeted intervention can prevent nearly 50% of DFU and amputation events (6).

Despite the well-recognized clinical severity of diabetic foot complications, systematic nursing competency frameworks and standardized early risk assessment systems for DF prevention remain insufficient. Current clinical practice still lacks a scientific, multidimensional, and targeted nursing assessment system for early DF risk identification. Against this background, this study aimed to construct a comprehensive early risk assessment system for high-risk diabetic feet. The findings are expected to clarify core nursing competencies for DF prevention and management, provide evidence for optimizing foot care training programs, and support standardized evaluation of nursing capabilities for diabetic foot prevention across different levels of medical institutions.

## 2 Methods

### 2.1 Establishment of the research team

The research team comprised four members, including one chief nursing officer, one nursing supervisor, one registered nurse, and one graduate nursing student. All members specialized in chronic disease management and held at least a bachelor’s degree. The team was responsible for drafting the initial version of the high-risk diabetic foot early risk assessment system, developing the expert consultation questionnaire, selecting consultative experts, and performing statistical analysis of expert ratings and feedback. The expert consultation questionnaire was developed based on a systematic literature review and established clinical guidelines for diabetic foot prevention and risk assessment.

### 2.2 Preliminary drafting of the early risk assessment system plan

Based on a comprehensive literature review and theoretical framework analysis, we developed the preliminary early diabetic foot risk assessment system and clarified relevant conceptual definitions. Literature retrieval was carried out in multiple databases including PubMed, Web of Science, CNKI, Wanfang Data, VIP and CBM with keywords such as high-risk diabetic foot, risk factors, diabetic foot, foot ulcer and nursing, and relevant policy documents and guidelines were also collected from the official website of the National Health Commission of China. Combined with the pathophysiological characteristics and early clinical indicators of diabetic foot, the preliminary framework of the assessment system was established covering three core domains of prevention, screening and management, and the finalized system was composed of six dimensions and 42 items.

### 2.3 Expert consultation

#### 2.3.1 Development of an expert consultation questionnaire

The expert consultation questionnaire was divided into three sections. The first was a cover letter elaborating the research background, objectives and research procedures to help experts fully comprehend this study. The second was the expert evaluation form, which included filling instructions, core concept definitions and all preliminary items of the early diabetic foot risk assessment system. All items were assessed for importance via a 5-point Likert scale, with scores from 1 (completely unnecessary) to 5 (completely necessary), and the intermediate options were 2 for unnecessary, 3 for moderately necessary and 4 for necessary. Meanwhile, blank fields were set for experts to propose item addition, deletion and revision opinions, so as to collect qualitative suggestions for further item improvement. The third part was the expert basic information questionnaire, which collected experts’ demographic information, professional experience, familiarity with the research theme as well as the empirical and theoretical basis for their evaluation.

#### 2.3.2 Selection of consultation experts

A total of 15 experts engaged in diabetic foot clinical practice were recruited for this expert consultation. The inclusion criteria were having at least five years of working experience in diabetic foot care, holding intermediate or above professional titles, and agreeing to participate in this study voluntarily.

#### 2.3.3 Expert consultation

The expert consultation was conducted between June and July 2024. The consultation questionnaires were distributed electronically via email, with a two-week response window. After collecting the first-round questionnaires, all data were systematically collated and analyzed. Based on the expert ratings, the mean importance score and coefficient of variation (CV) for each item were calculated. Items with a mean importance score below 3.5, a CV above 0.25 (7), or those for which experts proposed revisions or deletions were reviewed by the research team. Following group deliberation, items were either revised or removed, and the second-round consultation questionnaire was developed accordingly. The revised questionnaire was then sent to the expert panel for re-evaluation of item importance and further refinement suggestions. The consultation process was concluded once expert consensus was achieved.

### 2.4 Statistical methods

Data processing and statistical analysis were performed using SPSS 22.0. Categorical data are expressed as frequencies and percentages, while continuous data are presented as mean ± standard deviation. The expert activity coefficient, authority coefficient, and coordination degree were calculated, and the statistical significance of Kendall’s coefficient of concordance (Kendall’s W) was analyzed.

## 3 Results

### 3.1 Basic information about the experts

Among the 15 consulted experts, four were from Xi’an City, four from Hunan Province, and seven from Gansu Province. The experts’ age ranged from 34 to 56 years, with a mean age of 44.80 ± 7.78 years. In terms of professional working experience, one expert had 1–9 years of experience, eight had 10–19 years, and six had 20 years or above. Regarding educational attainment, one expert held a bachelor’s degree, six held master’s degrees, and the remaining eight experts received college education or professional vocational training. In terms of professional titles, six experts held senior professional titles and nine held intermediate professional titles. Thirteen experts possessed specialized nurse certification. In terms of professional fields, two experts specialized in diabetic foot and podiatry (including one physician and one specialized nurse), two engaged in orthopedic nursing, and three served as nursing administrators. Notably, several experts had multiple professional research orientations and clinical job responsibilities, as detailed in **Table 1**.

**Table 1:** Basic information form of experts.

No.	Professional title	Educational level	Work experience (Years)	Professional field
1	professor	Doctoral degree	29	Chronic Disease Management
2	Supervising nurse	Master’s degree	14	Chronic Disease Management
3	Supervising nurse	Master’s degree	18	Nursing management
4	Supervising nurse	Bachelor’s degree	14	Chronic Disease Management
5	Chief nurse	Master’s degree	20	Chronic Disease Management
6	Assistant researcher	Doctoral degree	16	Chronic Disease Management
7	Supervising nurse	Doctoral degree	16	Department of Trauma Repair
8	Deputy Director and Nurse	Master’s degree	11	Nursing management
9	Supervising nurse	Master’s degree	14	First aid nursing
10	Associate professor	Doctoral degree	13	Chronic Disease Management
11	Chief nurse	Doctoral degree	14	Critical care
12	Professor	Doctoral degree	30	Department of Orthopedics
13	Professor	Doctoral degree	29	Chronic Disease Management
14	Associate professor	Doctoral degree	20	Department of Orthopedics
15	Deputy Director and Nurse	Master’s degree	25	Chronic Disease Management

### 3.2 Expert engagement level

In this study, 15 consultation forms were distributed in the first round, with 11 valid responses returned, yielding an effective response rate of 73.3%. Among the respondents, six experts provided a total of 20 written recommendations. In the second round, consultation forms were distributed to the 11 experts who participated in the previous round. All 11 forms were returned and deemed valid, resulting in a 100.0% effective response rate. Five experts provided a total of seven written recommendations during this phase.

### 3.3 Expert authority level

In this study, during the first round of expert consultation, the coefficient of expert enthusiasm (Cs) was 0.806 and the authority coefficient (Ca) was 0.798. In the second round, the Cs and Ca values were 0.894 and 0.972, respectively. The Kendall’s concordance coefficients (Kendall’s W) for the two rounds were 0.802 and 0.933.

### 3.4 Degree of expert coordination

The degree of consensus among expert opinions is typically assessed using Kendall’s coefficient of concordance (Kendall’s W) and the coefficient of variation (CV). Following two rounds of consultation in this study, the coefficient of variation for the importance ratings of all items was below 0.05 in both rounds.

### 3.5 Consultation results of the early risk assessment system for diabetic foot

After the first round of expert consultation, the average importance score of all indicators was 3.92–5.00, and the coefficient of variation (CV) was 0.09–0.31. Indicators with CV ≥ 0.25 were excluded after group discussion by the research team, and nine new items were supplemented into the evaluation system.

In the neurological evaluation dimension, the original item “Have you ever felt numbness, tingling, or pain in your feet?” was revised to “Have you experienced foot numbness, tingling, pain, dry skin or cotton-wool-like walking sensation?”. In the vascular lesion assessment module, one item was optimized to improve clinical practicability without changing its core meaning: “Have you received lower extremity arteriovenous color Doppler ultrasound, ankle-brachial index detection, angiography, peripheral vascular Doppler examination, dorsalis pedis/posterior tibial artery palpation or skin temperature measurement and other related vascular examinations?”. Multiple items in the personal foot hygiene and care domain were revised for better clinical applicability. The toenail care item was adjusted from “Do you regularly trim your toenails and ensure they are smooth at the edges?” to “Do you trim toenails regularly to keep edges smooth without excessive trimming depth?”. The daily foot inspection item was revised into “Do patients or their family members conduct daily foot examinations to assess skin integrity, dorsalis pedis artery pulsation and ulcerative lesions?”. The foot skin moisture management item was revised to “Do you keep feet dry and control foot soaking time within 30 minutes?”. In terms of indicator supplementation, regular hypoglycemic drug use, blood glucose control level, diabetes-related knowledge, awareness of diabetic foot complications, combined chronic disease history and routine liver function monitoring were added as risk assessment indicators. Arteriosclerosis obliterans was separately listed in the macrovascular complication dimension. Foot thermal and tactile hypoesthesia assessment including tingling sensation was added, and the 128-Hz tuning fork test was incorporated for vibratory sensation evaluation. Rest pain was included in peripheral vascular complications, and the original vague term “vascular complications” was revised to “peripheral vascular complications” to enhance terminology specificity. Meanwhile, items concerning peripheral vascular color Doppler ultrasound examination and lower limb amputation history were deleted, and the item about long-term use of inappropriate footwear in daily foot care was further modified. Finally, the constructed early risk assessment system for diabetic foot was composed of 6 dimensions and 40 items after the first round of expert consultation.

Following the second round of expert consultation, the importance scores of all items ranged from 4.12 to 5.00, with coefficients of variation (CV) of 0 to 0.20, fully satisfying the preset screening criteria. In accordance with experts’ suggestions, the research team revised two items and supplemented two new items. Specifically, Charcot foot was added to the structural foot abnormality assessment subcategory within the foot deformity and injury module, and regular liver function monitoring was incorporated into the personal hygiene and foot care domain. After two rounds of expert consultation, consistent expert consensus was reached, and the consultation process was terminated. The final diabetic foot prevention and control nursing competency system consists of 6 dimensions and 42 items (**Table 2**).

**Table 2:** Early risk assessment system for high-risk diabetic foot.

First level entry	Secondary entry	Importance score ( $\bar{X}$ ± *S*)	Coefficient of variation	Full-score rate (%)
Assessment of Foot Deformities or Injuries	Charcot Foot: A condition characterized by the collapse of joint surfaces and deformation of the limb. Hallux Valgus: A foot deformity where the big toe deviates outward beyond the normal physiological angle (generally considered to be 15°)	5.31 ± 4.51	0.08	82
	Do you have a history of foot trauma or surgery	5.54 ± 4.45	0.10	73
	Is there a fungal infection	5.31 ± 4.51	0.08	82
Assessment of Personal Hygiene and Foot Care (7 items)	Do the patient or their family members check the foot condition daily, including the skin, dorsalis pedis artery pulsation, and any signs of ulceration	5.00	0	100
	Do you regularly trim your toenails and ensure the edges are smooth to prevent cutting them too deeply	5 ± 0.30	0.06	91
	Do you keep your feet dry and avoid soaking them in water for extended periods (no longer than 30 minutes)	5 ± 0.30	0.06	91
	Do you choose soft and breathable shoes and socks	5.31 ± 4.51	0.08	82
	Frequency of foot washing	5.05 ± 3.67	0.14	64
	Do you protect your feet according to the correct methods, such as avoiding local hot or cold stimulation and avoiding walking barefoot	5 ± 0.30	0.06	91
	Do you often wear shoes that don’t fit well	5.10 ± 3.45	0.09	73
Risk Factor Assessment (22 items)	age	5.00	0	100
	gender	5.31 ± 4.51	0.08	82
	educational level	5.31 ± 4.51	0.08	82
	Body Mass Index	5 ± 0.30	0.06	91
	duration of diabetes	5 ± 0.30	0.06	91
	hypertension	5.10 ± 3.45	0.14	82
Risk Factor Assessment	Hyperlipidemia	5.10 ± 3.45	0.14	82
	kidney disease	5.31 ± 4.51	0.08	82
	Retinopathy	5.31 ± 4.51	0.08	82
	waist circumference	5.04 ± 3.51	0.20	73
	history of foot ulcers	5 ± 0.30	0.06	91
	knowledge related to diseases and prevention	5.31 ± 4.51	0.08	82
	macrovascular disease	5.00	0	100
	history of other chronic diseases	5 ± 0.30	0.06	91
	smoking habit	5.31 ± 4.51	0.08	82
	drinking habit	5.06 ± 3.30	0.11	64
	exercise status	5 ± 0.30	0.06	91
	nutritional status	5 ± 0.30	0.06	91
	psychological status	5.31 ± 4.51	0.08	82
	Are hypoglycemic drugs taken regularly	5.00	0	100
	glycemic control status	5.00	0	100
	awareness of the dangers of diabetic foot	5 ± 0.30	0.06	91
Laboratory index assessment(3 items)	Whether regular checks are conducted for fasting blood glucose, 2-hour postprandial blood glucose, and glycated hemoglobin	5.00	0	100
	Whether regular checks are conducted for total cholesterol, triglycerides, high-density lipoprotein cholesterol (HDL-C), and low-density lipoprotein cholesterol (LDL-C)	5.55 ± 4.45	0.10	73
	Whether regular checks are conducted for the urine albumin-to-creatinine ratio (UACR) and glomerular filtration rate (GFR)	5.55 ± 4.45	0.10	73

## 4 Discussion

### 4.1 The early risk assessment system for high-risk diabetic feet is scientific and reasonable

This study was designed to establish a scientifically rigorous and systematically structured early risk assessment system for high-risk diabetic foot, and to evaluate its potential for practical clinical application. The Delphi expert consultation method was employed as the primary methodology, ensuring the accuracy and reliability of the findings through a standardized and iterative consensus-building process.

In Delphi consultation, expert selection is critical (8). This study meticulously selected experts with extensive theoretical and practical experience in diabetic foot care, orthopedics, and chronic disease management, all holding intermediate or higher professional titles and having over five years of clinical or research experience in related fields. This ensured the authority and representativeness of the expert panel. Two rounds of consultation were conducted, with response rates exceeding 50.0% in both rounds (9). The expert authority coefficients (Cr) reached 0.802 and 0.933, respectively, far exceeding the conventional threshold of 0.70 (10), indicating high confidence and authority in the consultation content. Coordination among expert opinions was favorable, with statistically significant concordance (P<0.05) and coefficients of variation (CV) <0.25 for all items, confirming high expert consensus and low evaluation dispersion.

These outcomes support the scientific rigor of the system, providing a robust foundation for healthcare institutions to refine prevention and management protocols according to local operational contexts.

### 4.2 The early risk assessment system for high-risk diabetic feet is comprehensive and targeted, with distinct advantages over existing tools

This study focused on early risk assessment of high-risk diabetic feet, aiming to develop a comprehensive, targeted preventive care system. Prevention of foot ulcers is cost-effective and clinically impactful (11), making early identification of at-risk patients a priority for nursing practice.

Compared with existing diabetic foot risk assessment tools, which often focus narrowly on neuropathy or vascular status, the present system demonstrates three key differences and strengths: (1) Multidimensional and holistic coverage: It integrates 6 dimensions and 42 items, spanning not only pathophysiological factors (neuropathy, vascular disease, foot deformities) but also behavioral and social determinants (foot self-care habits, health literacy, and metabolic control). This addresses a critical gap in many current tools, which overlook modifiable behavioral risk factors that are particularly relevant in the Chinese clinical context; (2) Early risk orientation: Unlike tools designed for advanced-stage ulcer risk prediction, this system emphasizes pre-ulcerative early risk screening, enabling timely intervention before irreversible tissue damage occurs; (3) Contextual adaptability: Developed with input from local experts, the system incorporates clinical scenarios common in Chinese patients, such as delayed medical consultation and limited foot self-care practices, making it more culturally appropriate than international tools that may not reflect regional healthcare realities.

Nurses can use this system to conduct targeted preventive assessments, including screening for foot deformities, fungal infections, skin integrity issues, and modifiable behavioral risks (12). Accurate screening enables early identification of high-risk individuals, timely diagnosis, and targeted interventions, thereby reducing the incidence of foot gangrene and amputation (13). Structured patient education, supported by the system’s clear risk stratification, can also enhance patients’ awareness of foot protection and risk factors, further improving outcomes (14).

### 4.3 Limitations and future development needs of the assessment system

While the system demonstrates strong methodological rigor and clinical relevance, several key areas require further development before widespread clinical implementation; (1) Prospective validation in large patient cohorts: The current study relied on expert consensus for item development. Future research will conduct a prospective longitudinal study to validate the system’s predictive performance (sensitivity, specificity, positive/negative predictive values) in a large sample of high-risk diabetic patients, establishing its ability to accurately predict foot ulcer development; (2) Risk stratification and cut-off value determination: Further analysis of the scale’s scoring distribution is needed to define clinical risk thresholds (e.g., low/medium/high risk) and corresponding management recommendations, to guide tiered clinical interventions; (3) Simplification for clinical practice: The 42-item system, while comprehensive, may be time-consuming for routine use. A follow-up study will explore the development of a short-form version by selecting high-priority items based on predictive validity, balancing comprehensiveness with clinical feasibility; (3) Cross-setting validation: The system requires validation across different healthcare settings (primary care vs. tertiary hospitals) and geographic regions to confirm its generalizability and identify setting-specific adjustments needed for broader adoption. Addressing these needs will strengthen the system’s clinical utility and ensure it can be reliably integrated into routine diabetic foot prevention and care pathways.

In summary, the present early risk assessment system for high-risk diabetic feet is scientifically rigorous, comprehensive, and contextually adapted to local clinical needs. With further prospective validation and refinement, it holds significant potential to support standardized early risk screening, guide targeted preventive interventions, and ultimately reduce the burden of diabetic foot complications.

## 5 Conclusions

Diabetic foot disease poses a major public health challenge and imposes substantial socioeconomic burdens. This study developed a scientifically rigorous and clinically rational early risk assessment system for high-risk diabetic foot populations. Integrating health education, risk factor evaluation, specialized screening, and wound assessment, the system defines stage-specific nursing competencies to support standardized preventive care in both specialty clinic and inpatient settings. A rigorously structured iterative Delphi process was adopted to ensure the reliability of the findings. The established system clarifies core nursing competencies, provides a solid foundation for the development of professional training programs, and offers nursing administrators a standardized framework for cross-setting competency evaluation and targeted intervention development. Clinically, it facilitates the early identification of high-risk populations, effective disease prevention, and controlled pathological progression, thereby reducing diabetic foot-related disability and mortality, improving patients’ quality of life, and alleviating associated societal burdens. Restricted by time and research resources, the expert panel was predominantly recruited from Gansu Province, which may limit the generalizability of the results. To enhance its applicability, the competency framework will be operationalized into a Likert-scale survey instrument for multi-regional empirical studies, enabling further system validation and refinement based on real-world clinical feedback and regional variations.

## Data Availability

The raw data supporting the conclusions of this article will be made available by the authors, without undue reservation.
